# Shaping the Future of DHT Assessment: Insights on Industry Challenges, Developer Needs, and a Harmonized, European HTA Framework

**DOI:** 10.3390/jmahp13030046

**Published:** 2025-09-04

**Authors:** Fruzsina Mezei, Emmanouil Tsiasiotis, Michele Basile, Ilaria Sciomenta, Elena Maria Calosci, Debora Antonini, Adam Lukacs, Rossella Di Bidino, Americo Cicchetti, Dario Sacchini

**Affiliations:** 1 The Graduate School of Health Economics and Management (ALTEMS), Università Cattolica del Sacro Cuore, 00168 Rome, Italy; emmanouil.tsiasiotis@unicatt.it (E.T.); michele.basile@unicatt.it (M.B.); ilaria.sciomenta@altemsadvisory.it (I.S.); elenamaria.calosci@altemsadvisory.it (E.M.C.); debora.antonini@altemsadvisory.it (D.A.); rossella.dibidino@policlinicogemelli.it (R.D.B.); americo.cicchetti@unicatt.it (A.C.); 2 EIT Health, 80807 Munich, Germany; adam.lukacs@eithealth.eu; 3 Fondazione Policlinico Universitario Agostino Gemelli IRCCS, Università Cattolica del Sacro Cuore, 00168 Rome, Italy; 4 Clinical Ethics Consultation Service, Fondazione Policlinico Universitario Agostino Gemelli IRCCS, Università Cattolica del Sacro Cuore, 00168 Rome, Italy; dario.sacchini@unicatt.it; 5 Research Centre for Applied Bioethics and Medical Humanities (CRiBCeMH), Università Cattolica del Sacro Cuore, 00168 Rome, Italy

**Keywords:** digital health technologies, digital health, market access, HTA, reimbursement, industry perspective, Europe

## Abstract

**Introduction:** Market access, pricing, and reimbursement of digital health technologies (DHTs) in Europe are significantly challenged by regulatory fragmentation and various assessment methodologies. Understanding the challenges and priorities of technology developers is essential for developing effective and relevant policy responses. This study explores perceived barriers and developer-driven priorities to inform the development of a harmonized health technology assessment (HTA) framework under the EDiHTA project. **Methods:** A mixed-methods approach was adopted, including a scoping review to identify key challenges, a survey of 20 DHT developers, and interviews and focus groups with 29 industry representatives from startups to multinational companies across 10 European countries during 2024. **Results:** Key challenges included a lack of transparency in reimbursement processes, fragmented HTA requirements, and misalignment between traditional evidence models and the agile development of DHTs. Developers highlighted the need to integrate real-world evidence, consider usability and implementation factors, and provide structured, lifecycle-based guidance. Financial barriers and procedural burdens were particularly significant for small and medium-sized enterprises. **Conclusions:** These findings highlight the need for an HTA framework that reflects the iterative nature of digital development, integrates real-world evidence, and reduces uncertainty for developers. The EDiHTA project aims to respond to these challenges by building a harmonized and flexible approach that aligns with the goals of the European HTA Regulation.

## 1. Introduction

The rapid evolution of digital health technologies (DHTs) presents significant opportunities for healthcare systems globally. These technologies, encompassing mobile health applications, telemedicine, and artificial intelligence (AI) solutions, have the potential to revolutionize disease management, improve patient outcomes, and enhance the efficiency of healthcare systems [1]. However, unlike pharmaceuticals and medical devices that follow well-established regulatory, health technology assessment (HTA), and reimbursement frameworks, the pathways applicable to DHTs are often not clearly defined or are still emerging in some countries. Their variety, broad range of use cases, software-driven iterative nature, and user-centricity pose significant challenges for conventional value assessment models [2,3,4]. The connection between market access, HTA, and pricing and reimbursement in relation to DHTs is evolving, with a growing emphasis on flexible and adaptive assessment methodologies that better align with the unique characteristics of DHTs. However, the methodological questions regarding the assessment of DHTs are still significant, which may limit their integration into health systems and delay access by healthcare professionals and patients.

To improve the uptake of DHTs, countries are taking a variety of approaches to integrate them into their national healthcare systems. Germany [5], France [6], and Belgium [7] have implemented comprehensive fast-track pathways coupled with funding streams that enable safe and effective DHTs to be reimbursed by national health insurance funds. Other countries like Finland [8], Spain [9], and the United Kingdom [10] have developed dedicated assessment methodologies for DHTs to inform decisions regarding implementation. Some countries, like France and the UK, utilize additional checklists for AI-enabled technologies that assess multiple additional factors in addition to the traditional HTA process [11,12]. The majority of countries still rely on the traditional HTA processes, making it more challenging for DHTs to demonstrate their value within existing frameworks due to a lack of flexibility to account for digital-specific characters like usability data, cybersecurity, or real-world performance [13].

The nature and complexity of digital health technologies, as well as the methodological limitations associated with their assessments, can contribute to uncertainties for health technology developers^1^ in both regulatory and HTA-informed decision making [14]. The lack of transparency in pricing and reimbursement policies further complicates the process, as many national systems do not provide clear guidelines on what constitutes sufficient evidence for coverage decisions, and pricing negotiations are confidential. As a result, some developers opt to launch their products in markets with more predictable and standardized pathways, such as the United States or China, rather than navigating Europe’s fragmented landscape [15]. Such delays in market access may limit the timely availability of innovative services for patients and health systems in the region. These challenges underscore the importance of understanding how system-level differences translate into practical barriers for developers. It also highlights the need for harmonized policies and streamlined HTA processes to facilitate market access and the reimbursement of DHTs across Europe [16,17], ensuring that technology developers can navigate this complex environment efficiently.

Recognizing these systematic challenges, the European Digital Health Technology Assessment (EDiHTA) project was launched in 2024, funded by the European Commission (Horizon Europe) for four years [18,19]. EDiHTA’s mission is to map and address current regulatory and HTA barriers by developing a harmonized, flexible, and transparent HTA framework tailored specifically to the unique characteristics of DHTs. This framework will enable the assessment of various DHTs—including artificial intelligence (AI) solutions—with different intended purposes, while also considering different maturity levels and decision-making levels. EDiHTA brings together 19 partners from 10 European countries, including policymakers, HTA bodies, developers, patient organizations, and academic institutions. The goal of the project is to harmonize different standards relevant to the assessment of DHTs, while considering the development of the framework and the perspectives of all relevant stakeholders. To this end, stakeholder involvement was embedded from the beginning, with participants contributing to the definition of key concepts, evidence requirements, and practical considerations through consultations and iterative feedback rounds.

For health technology developers, the establishment of a clear and predictable HTA pathway is crucial, as market access and reimbursement decisions directly influence their go and no-go decisions in their development strategies. Their investment decisions often hinge on whether a clear and predictable pathway exists for obtaining market access and funding at the national level [20]. It is therefore important to consider the developer perspective, as investment decisions are often influenced by whether clear assessment and reimbursement pathways are available. The successful integration of DHTs into healthcare systems requires collaboration among multiple stakeholders, including technology developers, healthcare providers, patients, policymakers/payers, and HTA agencies. Initiatives such as EDiHTA exemplify the importance of cross-sector and cross-border partnerships in addressing the challenges associated with market access and the reimbursement of DHTs. To support the design of future frameworks like EDiHTA, it is first necessary to understand the barriers, needs, and facilitators experienced by stakeholders, including developers, in navigating current assessment and reimbursement processes.

## 2. Objective

The aim of this research is to identify the specific challenges and requirements faced by technology developers in securing market access and reimbursement for DHTs in Europe. By identifying these barriers, this study aims to support the development of a harmonized HTA framework under the EDiHTA project that accounts for developers’ needs.

## 3. Methods

To identify the needs and requirements of technology developers, a threefold methodology was employed. A scoping literature review was conducted to identify and analyze the factors that act as barriers or facilitators in the decision-making processes of health technology developers. By examining existing evidence, this review identified key barriers and facilitators shaping decision-making, offering insights into what fosters sustained innovation or hinders technological advancements in healthcare. Based on the scoping review, a survey was developed and distributed among technology developers to identify the most critical factors in developing new digital health technologies from a developer’s perspective. Survey responses were followed up with focus groups and interviews with participating technology developers. Additional participants were identified and included in the interview process through a snowball sampling method, based on recommendations from stakeholders already involved.

The literature review was conducted as a scoping review (ScR), following the framework outlined in the PRISMA-ScR guidelines [21]. The search strategy aimed to balance comprehensiveness with precision, capturing a broad array of studies related to digital health technology assessment and decision-making while also ensuring the inclusion of only methodologically sound, peer-reviewed, and relevant publications. The core search string was developed to include terms related to the development, decisions, assessment, evaluation, and use of digital health technologies. The search was conducted in multiple electronic databases, including PubMed, Embase, Web of Science, and Scopus, supplemented by grey literature from policy organizations, regulatory agencies, and industry reports. Studies published in English between 2012 and 2024 were included to ensure that the analysis was both current and relevant. Inclusion criteria encompassed studies addressing aspects of the assessment, implementation, and use of digital technologies involving relevant stakeholders (e.g., clinicians, patients, providers). Exclusion criteria included duplicates, studies unrelated to the objectives, case reports, letters to the editor, and articles with incomplete information. After duplicate removal, an initial screening was conducted based on titles and abstracts. Eligible studies were then subjected to full-text reviews to confirm their relevance to the defined criteria. Two junior researchers independently screened all titles and abstracts in a double-blind manner. Disagreements regarding the inclusion of studies were resolved through discussion with a senior researcher, who made the final decision in cases of persistent uncertainty. The quality of the included studies in the scoping review was assessed using the Joanna Briggs Institute (JBI) critical appraisal tools [22]. The full search strategy, including the detailed query and the application of PRISMA-ScR methods, is presented in Supplementary File S1.

The questionnaire was created to complement the results of the literature review. The survey was distributed to international health industry players and start-ups/SMEs through conferences (e.g., HIMSS Europe 2024), networking events, social media, and consortium partner networks between June and September 2024 to understand what factors influence the decisions around the development of a DHT. Closed questions were used to capture the decision-making process regarding developers’ willingness to invest in the development of a DHT, as well as the main factors that influence their decision to go to market and other strategy-related issues. No open questions or qualitative-related questions were included. In exploring the priorities of digital health technology developers, the developers shared information regarding their company and background and rated the importance of various aspects for market access and reimbursement on a Likert scale from 1 to 9. For the survey, reliability and validity checks were conducted, including a pilot test before distribution: an initial version of the questionnaire was reviewed by a panel of experts, composed of academic researchers with experience in HTA that evaluated the clarity of the questions, their relevance to the study objectives, and the comprehensiveness of the domains covered; the questionnaire was tested on a preliminary sample of developers, representative of the target population. The feedback received was used to improve the wording of the questions and ensure consistency in the Likert response scale. The final version of the questionnaire demonstrated a good level of clarity and comprehensibility, and no systematic issues were reported during its completion. The questionnaire can be found in Supplementary File S2.

Focus groups and interviews were intended to validate the results of the literature review and survey, regarding the past and present, while also imagining the future of the DHT market in the EU and how the EDiHTA framework could provide a decision-making tool for all relevant stakeholders, including developers. Developers who completed the survey were invited to participate further in interviews and focus groups between September and November 2024. Additional participants were identified using a snowball sampling approach, whereby existing contacts recommended relevant peers or colleagues. A standardized protocol was developed for both focus groups and interviews (Supplementary File S3), with open-ended questions to guide the sessions. This approach allowed flexibility for discussions to cover various aspects and types of technologies. The protocol was structured based on the International Health Technology Assessment Model (IHTAM) [23], which guided participants through a reflective and forward-looking process. In the first part of the sessions, participants were asked to “learn from the past and present” by describing the current market access and reimbursement processes and identifying limitations within current HTA systems. In the second part, participants were encouraged to “imagine the future”, visualizing an ideal HTA process while identifying potential barriers and facilitators of its realization. Once challenges and needs were identified, stakeholders were asked to decide whether existing methods could be improved or if novel methods needed to be developed to address these challenges.

A standardized data extraction form was used to capture relevant information from the scoping review, survey, and qualitative data collection. The extracted data were synthesized using a thematic analysis approach to identify recurring themes across regulatory challenges, reimbursement models, HTA methodologies, and the impact of real-world data (RWD) on market access [24]. Thematic analysis of qualitative data followed an iterative coding process to ensure consistency and reliability. Interview and focus group sessions were audio- or video-recorded to ensure accuracy and take comprehensive notes. All participants were asked to validate the summary of their input following each session to ensure the accuracy and completeness of the responses collected. Results were analyzed through a thematic analysis process.

## 4. Results

### 4.1. Literature Review

**Figure 1:**
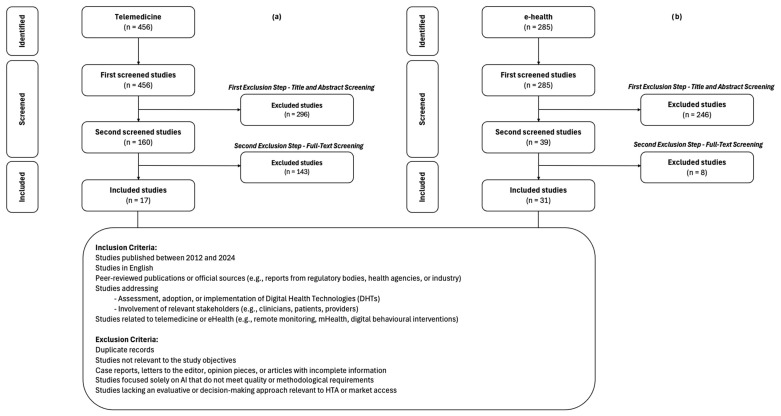
PRISMA-ScR flowchart of literature review results: (**a**) telemedicine; (**b**) eHealth.

A total of 1247 studies were initially identified through the database search, which underwent a two-stage screening process. After title and abstract screening, 705 studies were excluded due to irrelevance, leaving 542 studies for full-text review. The studies were categorized into three key thematic areas: telemedicine (456 studies), eHealth (285 studies), and artificial intelligence (AI) (5 studies). However, all AI-related studies were excluded due to a lack of alignment with the study criteria, leaving a final inclusion of 17 studies for telemedicine [25,26,27,28,29,30,31,32,33,34,35,36,37,38,39,40,41] (Figure 1a) and 31 for eHealth [42,43,44,45,46,47,48,49,50,51,52,53,54,55,56,57,58,59,60,61,62,63,64,65,66,67,68,69,70,71,72] (Figure 1b). The five studies identified in the field of AI were excluded as they did not provide relevant evidence in relation to the specific objective of this study: the identification of factors acting as barriers or facilitators in the development, implementation, and market access of DHTs by developers. In particular, the AI studies analyzed were primarily focused on technical or clinical aspects (e.g., diagnostic accuracy, algorithmic performance), without delving into the regulatory, economic, or systemic context implications that influence development and commercialization decisions.

The literature review identified key barriers and facilitators influencing the development and implementation of DHTs, particularly in the areas of telemedicine and eHealth (Table 1). For telemedicine, enhanced accessibility, diagnostic accuracy comparable to in-person visits, high patient satisfaction, and cost reduction were recognized facilitators. However, several barriers remained, such as digital literacy disparities among older adults and socio-economically disadvantaged groups, data security concerns, lack of interoperability with electronic health records (EHRs), and regulatory complexity due to inconsistent legal and reimbursement frameworks across countries. Similarly, eHealth solutions served as facilitators for improved chronic disease management, enhanced patient engagement, and cost savings through reduced healthcare utilization. Yet, their long-term sustainability was challenged by limited funding models, regulatory compliance requirements, and the absence of standardized evaluation frameworks.

Several key factors were identified that influence technology developers’ decisions on whether to continue or discontinue DHTs in the development phase. Regulatory and market access barriers, such as the lack of a harmonized European framework, create inconsistencies in approval and reimbursement, discouraging innovation. Financial constraints, including high compliance, certification, and proof generation costs, pose notable challenges, particularly for SMEs with limited resources. Technological issues, such as integration with existing health systems, interoperability, and cybersecurity concerns, hinder long-term adoption. Low user engagement, due to usability issues, gaps in digital literacy, and skepticism on the part of patients and healthcare providers, further affects the sustainability of DHTs.

The barriers and facilitators identified in the literature review, such as regulatory fragmentation, financial constraints, and technological integration, guided the development of the survey, allowing us to quantitatively assess the extent to which these challenges are perceived by technology developers themselves.

### 4.2. Survey

The survey was completed by 23 technology developers, with three incomplete responses that were excluded from the analysis. Responses were deemed incomplete if the respondent only completed part (1) General information of the survey, leaving part (2) Information regarding market access & reimbursement strategies empty. Out of the twenty technology developers with complete answers, six were categorized as mobile app developers, four as telemedicine developers, and five as AI developers. The other five developers were large industry representatives and/or part of MedTech Europe.

The majority of developers (*n* = 12/20) reported having a department or employee dedicated to evaluation, market access, or HTA within their organization. Additionally, 16 developers indicated that they investigate the HTA process of the target market before making a development decision. Out of the 31 factors grouped in seven main groups, developers prioritized clinical relevance, regulatory compliance, adaptability, and data privacy when designing and deploying DHTs. Ensuring alignment with clinical needs and patient populations was key, as was navigating regulatory landscapes for market access. Cost considerations were indicated as moderately important, with the importance of initial hardware costs rated as low but ongoing maintenance and financial sustainability, especially for AI and mobile apps, being significant. Factors related to direct costs, such as initial hardware expenses or shipping costs, received the lowest mean scores across all technology types. In contrast, domains such as regulatory compliance, usability, and stakeholder engagement consistently ranked higher, suggesting that non-financial barriers are more prominent concerns for developers. Additionally, usability, patient acceptance, and early clinician involvement are crucial for adoption, while data privacy remains of high importance in the implementation of DHTs, given Europe’s strict regulations. Aggregated results of the questionnaire as per type of technology can be found in Table 2. To contextualize the aggregated results, mobile apps in the sample included tools designed to support self-management and patient engagement in the form of mobile applications. Telemedicine technologies covered platforms for remote consultations and digital care coordination, while AI solutions ranged from diagnostic support tools to risk prediction algorithms integrated into clinical workflows.

Building on these quantitative insights, the subsequent focus groups and interviews provided an opportunity to validate and expand upon the survey findings, particularly regarding developers’ experiences with regulatory inconsistency, cost burdens, and the need for clearer assessment pathways.

### 4.3. Interviews and Focus Groups

Several themes raised during the focus groups and one-on-one interviews mirrored survey responses, reinforcing key priorities such as regulatory clarity, the impracticality of traditional evidence models, and the financial sustainability of assessment processes. A deeper context for these trends was provided through four focus group discussions with startups and SMEs and one focus group and five one-on-one interviews with industry representatives conducted between September and November 2024, involving, altogether, 29 participants in this phase of the research. In total, this study captured input from 31 unique digital health companies across Europe (see Table 3).

Developers confirmed that the main issues were navigating the varied and often unclear pathways for market access and reimbursement in different European countries. While obtaining a CE-mark may be sufficient for regulatory compliance, it does not guarantee market access or reimbursement for DHTs, as each country has its own set of requirements and pathways. In most European countries, there are no established processes in place to secure funding for digital health technologies. While securing a CE-mark is a clear advantage when entering the market, even after certification, national-level reimbursement processes and evidence requirements lack transparency, particularly in countries where reimbursement frameworks specific to DHTs are still evolving. As a potential strategy, some developers take a proactive, bottom-up approach by attempting to trigger decisions from payers to adopt their technologies or pursue an alternative route by selling their solutions directly to hospitals, utilizing this purchase model to achieve market entry. In countries like Germany, France, and Belgium, where a dedicated assessment framework for DHTs is implemented and coupled with reimbursement, several types of DHTs are not eligible to apply for reimbursement through the dedicated pathways (e.g., diagnostic tools, non-patient-facing tools, solutions that improve workflow). Developers feel that markets are currently cherry-picking DHTs, when the greatest added value would be in primary and secondary prevention.

Industry representatives and startups/SMEs highlighted the lengthy process of securing reimbursement, which can take up to 18 months in some countries. The lengthy processes in a field of ever-evolving innovation place considerable strain on the resources of developers, particularly for start-ups and smaller companies. Financial concerns were a major focus, particularly the high costs of clinical validation and compliance. Developers noted that initial investments in certification and compliance with both EU-wide and local regulations were substantial. One participant developing an AI solution mentioned that after investing heavily in trials to prove the value of their technology, slow reimbursement processes (ca. 18 months) in Germany nearly halted their market entry. Unlike larger multinational corporations, which may have diversified portfolios and greater financial resilience, smaller firms often rely on limited funding rounds. If initial efforts are unsuccessful, securing further investment can be challenging, leading to potential project termination or even bankruptcy. This unfavorable regulatory environment may also incentivize companies to shift focus toward markets with simpler, more predictable regulatory requirements, such as the US or China.

Developers highlighted the need for market access strategies that account for diverse regulatory environments when entering new countries. Compliance with regulations, especially those beyond core EU mandates like the General Data Protection Regulation (GDPR) [73] and European Health Data Space (EHDS) [74], was seen as a significant barrier. Different interpretations of regulations across countries made scaling products across Europe difficult. Developers described the challenge of needing a country-by-country strategy for access and reimbursement and often managing contradictory advice from consultants due to the complex regulatory landscape, resulting in delays and increased costs.

Consistent with literature and survey results, participants highlighted the limitations of current evidence generation models and advocated for the inclusion of real-world evidence (RWE), underscoring a shared industry preference for flexible, adaptive assessment models. Developers emphasized the limitations of RCTs, which are largely influenced by pharmaceutical practices and not well-suited for DHTs. Traditional RCTs were seen as too costly and time-consuming for digital health innovations. Industry representatives validated the findings of the literature review that classic studies, while valuable for drugs, are often impractical to design and implement for DHTs. They proposed alternative methods, such as simulation environments and platform studies, which provide faster and more accurate insights into the benefits of DHTs. Developers advocated for increased incorporation of RWE to complement traditional data collection models, allowing for continuous validation and adaptability throughout a DHT’s lifecycle.

When it came to evidence requirements, developers highlighted the limitations of traditional assessment domains for several types of DHTs and suggested looking at additional aspects more in line with the characteristics of DHTs. Apart from the classic assessment factors, usability and social acceptance should be at the center of the assessment process to ensure retention and greater patient benefits. AI developers also advocated for the importance of being transparent about the datasets that are used to train AI technologies, since data obtained based on a specific population/dataset may not be transferable to another product. A suggestion was to include assessment aspects related to the characteristics of the development (e.g., transparency with data sources).

When it came to the adaptability and scalability of DHTs, flexibility in development was mentioned as a critical factor. Integration into various clinical workflows and existing systems often presents underestimated challenges for developers. Mobile app developers stressed the importance of designing technology to align with distinct clinical environments, noting that this can differ widely across European healthcare systems. Interoperability was also highlighted as a significant barrier. Developers often prioritized markets with robust digital infrastructures, which led to difficulties in scaling technologies to regions with lower digital readiness and exacerbated access inequities. It was emphasized that to test and incorporate new evidence generation methods (e.g., RWD collection), infrastructures need to have adequate infrastructure to support these methods, which is often not the case in Europe.

**Figure 2:**
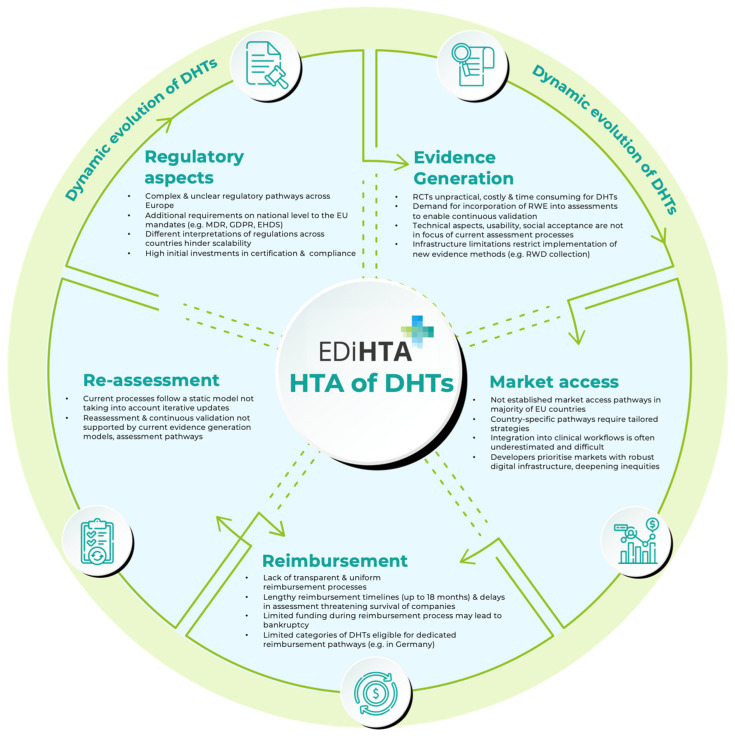
Challenges and barriers for developers along the lifecycle of DHTs. The figure does not aim to represent all lifecycle phases but rather offer a high-level overview of the main stages where developers typically face challenges and barriers. Arrows indicate the progression of DHTs over the lifecycle phases.

Strongly resonating with the results of the literature review, developers validated the need for broader stakeholder engagement throughout the HTA process, from development to post-market surveillance. This involvement would help ensure that DHTs meet real-world clinical needs and align with existing care pathways. Telemedicine developers highlighted the benefits of early clinician involvement in usability testing, which improved integration into healthcare settings and adoption rates. These perspectives not only highlight challenges across the lifecycle of digital health technologies but also point to broader limitations in current HTA practices, which are further explored in the following discussion. Key challenges and barriers for developers are summarized in Figure 2.

Across all research phases, developers consistently expressed that a harmonized HTA framework, such as the one envisioned by EDiHTA, would significantly help overcome existing challenges in market access and reimbursement. Participants viewed EDiHTA as a valuable framework to streamline evidence generation, reduce regulatory uncertainty, and facilitate early engagement with stakeholders. In addition, resources like interactive guidance platforms, standardized assessment pathways, and case study repositories were highlighted as key enablers for more efficient and predictable market entry.

## 5. Discussion

Developing and launching digital health technologies in Europe comes with a unique set of challenges, primarily due to the fragmented nature of health systems and regulatory frameworks. Developers consistently pointed to unclear, inconsistent, and duplicative requirements between existing HTA frameworks in European countries, complicating efforts to bring innovative digital solutions to market. One of the central issues highlighted was the absence of standardized and transparent HTA requirements across countries. Participants repeatedly stressed that without alignment and clarity, administrative and financial burdens will continue to hinder innovation. In addition to fragmented and inconsistent frameworks, developers raised broader concerns about several systemic challenges, including the inconsistent application and timing of evaluations across countries, limited stakeholder involvement, and static HTA models that are misaligned with the iterative nature of digital innovation. Infrastructure shortcomings and the high compliance burden, especially for SMEs, were also seen as major issues. These findings suggest that traditional HTA processes may not be sufficiently agile to accommodate DHTs and reinforce the need for a more dynamic, modular approach, affirming the relevance of EDiHTA in addressing developers’ operational and strategic needs across Europe. Similar findings have been reported by Mathias et al. [75] and van Kessel et al. [3], who emphasized that current assessment and reimbursement processes are often misaligned with the needs of developers and the nature of DHTs. Our study adds value by being one of the few that directly involved developers, providing first-hand insights into these barriers.

Incorporating RWE into assessments was highlighted as a key priority, accommodating the rapid, iterative development cycles of digital technologies. Several developers advocated for continuous data collection during the deployment of DHTs in the post-marketing phase to inform adaptive re-assessments, citing RWE as essential for this approach. To shorten market access and reimbursement timelines, the framework should allow DHTs to be evaluated at different development stages. Early and ongoing stakeholder discussions, along with clear guidance on evidence generation methods at each lifecycle stage, could further support developers. Timely consultations throughout the HTA process must be available. Establishing a pool of experts available for consultation, alongside HTA body consultations, could help accelerate market entry and reimbursement decisions.

In terms of the format and tools of the EDiHTA framework, developers indicated practical, structured guidance formats and dynamic toolkits as offering the best added value. A flexible, centralized resource could simplify compliance navigation, minimizing trial-and-error approaches and reducing development delays. Importantly, developers emphasized the perceived benefit of such a platform being tailored to reflect evolving digital health regulations, facilitating real-time adaptation of their strategies. To foster transparency in processes, the creation of a case study repository of already conducted assessments was seen as a valuable reference for developers for study types, sample sizes, outcomes, and other aspects. This would offer deeper insights into navigating the HTA landscape and highlight successful strategies employed by similar technologies. Case studies showing successful navigation of HTA processes were seen as practical references that developers could consult. For the uptake and sustainability of the EDiHTA initiative, the framework needs to be validated through pilot programs involving key European countries to test the methodology as well as multi-stakeholder engagement. From the developers’ perspective, these tools not only assist in understanding market expectations and streamlining planning processes. As Schliess et al. [76] noted in the case of DiGA, even with structured fast-track models in place, many types of DHTs remain excluded, reinforcing the need for broader, inclusive, and flexible frameworks like EDiHTA.

Our findings illustrate that developers are not only aware of these systemic inefficiencies but are actively seeking out practical ways to overcome them. They recognize that their concerns need a broader, coordinated, cross-border response rather than individual solutions. Across different company sizes and European markets, participants welcomed the initiative and expressed interest in actively engaging with EDiHTA, provided it aligns with their day-to-day realities and emerging needs. Ultimately, developers perceive the EDiHTA framework as a potential enabler of a coordinated approach to HTA of DHTs that promotes wider, faster adoption of DHTs in the European market. These insights reinforce the relevance of the EDiHTA initiative and confirm industry readiness to utilize and engage with the framework throughout the design and implementation phases.

This study has some limitations. Although participants represented a diverse mix of company sizes and geographic contexts, the sample size was relatively small (*n* = 31) and may have overrepresented companies already engaged in HTA or market access discussions. While feedback was collected and reviewed across different types of technologies, no substantial differences were observed; developers shared similar concerns across modalities, particularly around evidence requirements, financial constraints, and regulatory fragmentation. It should also be acknowledged that this study did not capture the perspectives of companies that may have exited the market or gone bankrupt due to these barriers. Including such experiences could potentially reinforce the findings or reveal even more critical challenges in navigating the current system. Future studies could explore whether more granular differences emerge in specific subgroups or use cases.

## 6. Conclusions

The literature review and perspectives from 31 unique DHT companies (20 companies in the survey, 29 companies in the qualitative phase, and 16 companies only participating in the interview phase), spanning startups, and well-established international firms across 10 European markets shed light on the needs of developers and the perceived added value of the EDiHTA framework in response to market access and reimbursement challenges, operational barriers, and decision-making processes when developing DHTs. The findings underscore the need for a standardized HTA methodology that aligns with the realities faced by technology developers in Europe. By aligning with the unique characteristics of DHTs, such a framework can support developers in navigating complex processes, driving the adoption of innovative technologies and ultimately improving patient outcomes across Europe.

Health technology developers suggested several critical areas that they believe the EDiHTA framework should prioritize. First, the simplification, clarification, and harmonization of regulatory and Health Technology Assessment (HTA) requirements are essential to reducing administrative burdens and accelerating market access and reimbursement pathways. A structured approach to linking assessment requirements with specific technology types could further enhance transparency and efficiency. Additionally, a multi-phase assessment model that integrates real-world evidence (RWE) into evaluations is crucial. Enabling digital health solutions to be assessed at different development stages with continuous data collection for adaptive (re-)assessment would facilitate a more dynamic and evidence-driven regulatory process. Financial sustainability remains a pressing concern, particularly for startups and SMEs, emphasizing the need for tailored funding opportunities to support data collection, clinical validation, compliance, and reimbursement. Finally, promoting transparency through a public HTA case study repository could serve as a valuable reference for developers, offering insights into study designs, sample sizes, key outcomes, and best practices of already conducted HTA processes.

Although HTA is primarily targeted at decision-makers, it concerns a wide range of stakeholders as it has an influence on the access and timing of the availability of new technologies to patients. Hence, it regards those who decide upon the availability of the technology on the market (e.g., policy makers), the end-users (e.g., patients), the ones prescribing the technologies (e.g., health care professionals), organizations reimbursing the technology (e.g., social security system, private insurance companies), and those that develop and sell the technology (e.g., industry). The foundational insights retrieved in this research will guide the iterative development and conceptualization of the EDiHTA framework, ensuring its sustainability and relevance in a dynamic digital health ecosystem.

While enabling innovation is a key driver, the framework will also need to support balanced, evidence-informed decision-making that reflects the perspectives of other stakeholders, including policymakers, payers, HTA bodies, clinicians, and patients. By addressing these multiple dimensions, EDiHTA can contribute to a more structured, transparent, and predictable environment for digital health technologies in Europe. Relevant stakeholder groups, including health technology developers, are part of the development phase as well and will be consulted in different phases of the EDiHTA project to review and evaluate designs.

## Figures and Tables

**Table 1:** Key barriers and facilitators of eHealth (eH) and telemedicine (T) solutions during development and implementation based on the literature review.

Factor	Barrier/Facilitator	Description	Lifecycle Phase
Regulatory Complexity (T)	Barrier	Different countries have varying regulations and reimbursement policies, creating access challenges. Variability in legal frameworks across countries creates barriers to market access.	Development and Implementation
Digital Literacy Disparities (T)	Barrier	Older adults and socioeconomically disadvantaged populations face difficulties in using telemedicine tools.	Implementation
Data Security Concerns (eH/T)	Barrier	Ensuring privacy and protection of patient data is a major challenge for telemedicine and eHealth platforms.	Development and Implementation
Integration with Electronic Health Records (T)	Barrier	Telemedicine systems often lack seamless interoperability with existing healthcare IT infrastructures.	Implementation
Enhanced Patient Engagement (eH)	Facilitator	eHealth tools empower patients by enabling self-monitoring and personalized health interventions.	Implementation
Improved Disease Management (eH)	Facilitator	Wearable sensors and real-time monitoring contribute to better disease outcomes.	Implementation
Cost Reduction (eH)	Facilitator	Digital interventions reduce healthcare utilization and improve efficiency in care delivery.	Implementation
Sustainability Issues (eH)	Barrier	The long-term adoption of eHealth technologies is hindered by funding gaps and a lack of reimbursement models.	Implementation
Financial Constraints (eH/T)	Barrier	High investment requirements make it difficult for startups to sustain projects.	Development and Implementation
Technological Challenges (eH/T)	Barrier	Interoperability and cybersecurity concerns hinder widespread implementation.	Development and Implementation
User Adoption (T)	Barrier	Low engagement from patients and providers reduces the feasibility of long-term success.	Implementation

**Table 2:** Results of survey to question “how important is [below factor] in your decision to develop and implement a technology on a scale of 1 (not important) to 9 (very important)?”.

	Aspects	Mobile App		Telemed		AI		All	
		*n* = 6		*n* = 4		*n* = 5		*n* = 20	
		M *	Md ** (Range)	M	Md (Range)	M	Md (Range)	M	Md (Range)
General aspects	Clinical need and target population	8.4	9 (6–9)	8.0	8 (7–9)	7.6	8 (6–9)	8.2	9 (6–9)
	Size of the target population	7.8	9 (5–9)	7.5	7.5 (6–9)	7.2	8 (5–8)	7.5	8 (5–9)
	Regulatory compliance requirements	7.4	7.5 (5–9)	8.8	9 (8–9)	8.0	8 (6–9)	8.2	8 (5–9)
	Complexity and time required	8.2	8.5 (5–9)	7.0	7 (5–9)	7.0	7 (5–9)	7.4	7.5 (4–9)
	Clear standard of care (SoC) comparator	8.4	8.5 (5–9)	7.0	7.5 (5–8)	7.3	8 (4–9)	7.6	8 (4–9)
Adaptability	Adaptability of the technology initially	8.2	8 (5–9)	7.0	6.5 (6–9)	7.2	7 (6–8)	7.4	7 (6–9)
	Adaptability in the development phase	7.0	6.5 (4–9)	7.8	7.5 (7–9)	7.2	8 (4–9)	7.3	7.5 (4–9)
	Adaptability in the use phase	7.8	8.5 (4–9)	8.5	8.5 (8–9)	8.0	9 (4–9)	8.3	9 (4–9)
Costs	Hardware maintenance (shipping cost, spare parts)	2.4	1 (1–7)	4.3	4.5 (1–7)	2.6	1 (1–5)	3.4	2 (1–7)
	Software development/bug maintenance	7.2	8 (4–8)	7.0	7 (5–9)	7.0	7 (5–9)	7.2	7 (4–9)
	Personnel needs (quantity and training)	7.4	7 (1–9)	6.5	6 (5–9)	7.6	8 (5–9)	6.8	7 (5–9)
	Other initial costs	7.5	7.5 (1–9)	3.5	3.5 (1–6)	3.5	3.5 (1–6)	5.5	6 (1–9)
	Ongoing device/hardware maintenance	4.4	3.5 (1–7)	5.0	6 (1–7)	2.6	1 (1–5)	4.1	5 (1–7)
	Ongoing software development/bug maintenance	6.4	6.5 (5–9)	6.5	6 (6–8)	6.4	7 (4–9)	6.6	6 (4–9)
	Ongoing personnel needs	7.2	6.5 (1–9)	7.3	7.5 (6–8)	7.2	9 (4–9)	6.8	7 (4–9)
	Other ongoing maintenance costs	7.0	6 (1–8)	8.0	8 (8)	2.7	1 (1–6)	5.1	6 (1–9)
Clinical outcomes	Direct impact on clinical outcomes	8.0	9 (4–9)	8.0	8.5 (6–9)	6.6	8 (1–9)	7.7	8.5 (1–9)
	Integration of care	7.6	8 (1–9)	8.3	8.5 (7–9)	8.2	9 (6–9)	7.6	9 (4–9)
	Continuity of care (long-term)	7.0	6.5 (1–9)	7.5	7 (7–9)	6.2	6 (3–9)	6.8	7 (3–9)
	Type of clinical improvement evidence	7.3	7 (1–9)	7.0	6.5 (6–9)	7.4	8 (5–9)	7.2	8 (4–9)
Market access, reimbursement	Market access	7.4	8 (3–9)	7.8	8.5 (5–9)	7.6	9 (5–9)	7.7	9 (3–9)
	Health Technology Assessment process	7.8	7.5 (6–9)	7.3	7.5 (6–8)	7.4	7 (6–9)	7.2	7 (2–9)
	Reimbursement and market access policies	7.6	9 (3–9)	7.5	8 (5–9)	7.0	7 (4–9)	7.7	8.5 (3–9)
	Expected return on investment (ROI)	8.0	9 (5–9)	6.5	6.5 (6–7)	8.4	8 (8–9)	8.1	8.5 (5–9)
Readiness of health systems and target populations	Readiness of health systems	7.6	9 (4–9)	7.8	7.5 (7–9)	7.6	8 (7–8)	7.7	8 (4–9)
	Readiness of patients: usability	8.6	9 (7–9)	8.0	8.5 (6–9)	8.0	9 (6–9)	8.2	9 (6–9)
	Readiness of patients: acceptance, willingness to use	8.4	9 (7–9)	7.5	7.5 (6–9)	8.0	8 (7–9)	8.1	8 (6–9)
	Readiness of patients: expected adherence	8.6	9 (8–9)	6.8	6 (6–9)	7.8	8 (7–9)	7.9	8 (6–9)
	Early involvement of patients/caregivers	7.8	7.5 (6–9)	7.0	7 (6–8)	7.8	8 (6–9)	7.6	8 (5–9)
	Early involvement of clinicians/HCPs	8.4	9 (6–9)	7.5	7.5 (6–9)	7.4	9 (3–9)	8.0	9 (3–9)
Data aspects	Privacy and security of the end user	8.4	9 (7–9)	7.3	7.5 (6–8)	8.4	9 (7–9)	8.4	9 (6–9)
	Data ownership	8.6	9 (7–9)	5.8	5.5 (5–7)	7.8	9 (6–9)	7.9	9 (6–9)
	Data privacy concerns (e.g., GDPR compliance)	8.6	9 (8–9)	7.3	7 (7–8)	8.2	9 (6–9)	8.4	9 (6–9)

* Mean. ** Median.

**Table 3:** Characteristics of developers participating in the survey and interview phase of this research.

Type of Interview	Survey	Type of Technology	Country of Main Market	Established
Focus Group: Mobile App	Yes	mHealth	Italy	2021
Focus Group: Mobile App	Yes	mHealth	Germany	2018
Focus Group: Mobile App	Yes	mHealth	France, Belgium, UK	2020
Focus Group: Mobile App	No	mHealth	Germany	2019
Focus Group: Mobile App	No	mHealth, Telemed	Germany	2017
Focus Group: Mobile App	No	mHealth	France	2017
Focus Group: Telemed	Yes	mHealth, Telemed	Austria	2015
Focus Group: Telemed	Yes	Telemed, AI	Ukraine, Germany	2019
Focus Group: AI	Yes	AI	US	2002
Focus Group: AI	Yes	mHealth, AI	Poland	2021
Focus Group: AI	No	AI	France	2020
Focus Group: Mixed	No	mHealth, Telemed	Finland	2020
Focus Group: Mixed	No	Robotics	Finland	2008
Focus Group: Mixed	No	mHealth	Finland	2020
Focus Group: Mixed	Yes	mHealth, Telemed	France, Germany, UK	2000
Focus Group: Mixed	Yes	mHealth, AI	Austria	2019
Focus Group: Mixed	No	AI	Austria	2018
Focus Group: Mixed	Yes	mHealth	France	2016
Interview	Yes	mHealth, Telemed, AI	Global	1896
Interview	Yes	Telemed	Germany	2000
Interview	Yes	mHealth, Telemed, AI	Global	1888
Interview	Yes	mHealth, Telemed, AI	Global	1888
Interview	No	mHealth, Telemed, AI	UK	NA
Focus Group: MedTech Europe	No	mHealth, Telemed, AI	Global	1989
Focus Group: MedTech Europe	Yes	mHealth, Telemed, AI	Global	1949
Focus Group: MedTech Europe	Yes	mHealth, Telemed, AI	Global	1896
Focus Group: MedTech Europe	No	mHealth, Telemed, AI	Global	1891
Focus Group: MedTech Europe	No	mHealth, Telemed, AI	Belgium	NA
Focus Group: MedTech Europe	No	mHealth, Telemed, AI	Belgium	NA
Did Not Participate	Yes	mHealth	France	2019
Did Not Participate	Yes	AI	Spain	2021
Did Not Participate	Yes	mHealth	Poland, Germany	2017
Did Not Participate	Yes	Telemed	France	2016

## Data Availability

The original contributions presented in this study are included in the article. Further inquiries can be directed to the corresponding author.

## Note

1 The developer is intended to be a manufacturer * or provider ** (if the DHT is an AI system), according to EU regulations. * Regulation (EU) 2019/1020: Art.3(8) ‘manufacturer’ means any natural or legal person who manufactures a product or has a product designed or manufactured and markets that product under its name or trademark. ** Regulation (EU) 2024/1689: Art.3(3) ‘provider’ means a natural or legal person, public authority, agency, or other body that develops an AI system or a general-purpose AI model or that has an AI system or a general-purpose AI model developed and places it on the market or puts the AI system into service under its own name or trademark, whether for payment or free of charge.)

## Supplementary Materials

The following supporting information can be downloaded at: https://www.mdpi.com/article/10.3390/jmahp13030046/s1, File S1. Search String for Literature Review. File S2. Survey. File S3. Focus Group and Interview Protocol

## Abbreviations

The following abbreviations are used in this manuscript:

DHT	Digital Health Technology
HTA	Health Technology Assessment
HTA-R	Health Technology Assessment Regulation
EHDS	European Health Data Space
GDPR	General Data Protection Regulation
RWE	Real-World Evidence
RCT	Randomized Controlled Trial
EHR	Electronic Health Record
