# Supplementary material for: Shaping the Future of DHT Assessment: Insights on Industry Challenges, Developer Needs, and a Harmonized, European HTA Framework
Source: J Mark Access Health Policy. 2025 Sep 4;13(3):46. doi: 10.3390/jmahp13030046 (PMC12452449; doi:10.3390/jmahp13030046)
Supplement: Supplementary file 1 [file jmahp-13-00046-s001.zip › jmahp-3709959-supplementary.pdf]

File S1. Search String for Literature Review

((((((((((Develop*)) AND (Heatlh ADJ2 Technology ADJ2 Assesment) AND (Decision* ADJ2 making)) OR (Decid*)) OR (use)) OR (assess*)) OR (evaluat*)) AND (Digital Health Technolog*)) AND (ffrft[Filter])) AND ((“systematic”[filter] OR “integrative research”[tiab] OR “integrative review*”[tiab] OR “integrative overview*”[tiab] OR “research integration*”[tiab] OR “research overview*”[tiab] OR “collaborative review*”[tiab] OR “collaborative overview*”[tiab] OR “systematic review”[pt] OR “systematic reviews as topic”[mh] OR “systematic review*”[tiab] OR “technology assessment*”[tiab] OR “technology overview*”[tiab] OR “technology appraisal*”[tiab] OR “Technology Assessment, Biomedical”[mh] OR HTA[tiab] OR HTAs[tiab] OR “comparative efficacy”[tiab] OR “comparative effectiveness”[tiab] OR “outcomes research”[tiab] OR “indirect comparison*”[tiab] OR “Bayesian comparison”[tiab] OR ((“indirect treatment”[tiab] OR “mixed-treatment”[tiab]) AND comparison*[tiab]) OR Embase*[tiab] OR Cinahl*[tiab] OR “systematic overview*”[tiab] OR “methodological overview*”[tiab] OR “methodologic overview*”[tiab] OR “methodological review*”[tiab] OR “methodologic review*”[tiab] OR “quantitative review*”[tiab] OR “quantitative overview*”[tiab] OR “quantitative synthes*”[tiab] OR “pooled analy*”[tiab] OR Cochrane[tiab] OR Medline[tiab] OR Pubmed[tiab] OR Medlars[tiab] OR handsearch*[tiab] OR “hand search*”[tiab] OR “meta-regression*”[tiab] OR metaregression*[tiab] OR “data synthes*”[tiab] OR “data extraction”[tiab] OR “data abstraction*”[tiab] OR “mantel haenszel”[tiab] OR peto[tiab] OR “der-simonian”[tiab] OR dersimonian[tiab] OR “fixed effect*”[tiab] OR “multiple treatment comparison”[tiab] OR “mixed treatment meta-analys*”[tiab] OR “umbrella review*”[tiab] OR ((“multiple paramet*”[tiab]) AND (“evidence synthesis”[tiab])) OR ((“multi-paramet*”[tiab]) AND (“evidence synthesis”[tiab])) OR ((multiparameter*[tiab]) AND (“evidence synthesis”[tiab])) OR “Cochrane Database Syst Rev”[Journal] OR “health technology assessment winchester, england”[Journal] OR “Evid Rep Technol Assess (Full Rep)”[Journal] OR “Evid Rep Technol Assess (Summ)”[Journal] OR “Int J Technol Assess Health Care”[Journal] OR “GMS Health Technol Assess”[Journal] OR “Health Technol Assess (Rockv)”[Journal] OR “Health Technol Assess Rep”[Journal]) AND (ffrft[Filter]))) AND (ffrft[Filter]))

File S2. Survey

EDiHTA: The first European Digital HTA framework co-created by all stakeholders along the value chain.

EDiHTA stands for the first European Digital Health Technology Assessment framework co-created by all stakeholders along the value chain, and is the title of a new research and innovation project recently funded with EUR 8 million under the EU’s Horizon Europe framework. Coordinated by the Università Cattolica del Sacro Cuore (Rome, Italy) and co-led by Prof. Americo Cicchetti and Prof. Dario Sacchini, the project successfully commenced its mission to develop a new, digital Health Technology Assessment (HTA) framework for Europe’s health ecosystem on 1 January 2024.

Digital Health Technologies: (Un)-locked potential

In a world where health systems worldwide are under constant pressure to provide high-quality services, digital health technologies (DHTs) like telemedicine, mHealth apps, or AI-based tools emerge as potential game-changers.

Digital health technologies are expected to improve both the quality and delivery of healthcare services while ensuring the sustainability of Europe’s healthcare systems. DHTs are also able to collect real-world data and evidence relevant to decision-makers.

However, existing Health Technology Assessment methodologies are unable to capture the real added value of DHTs. The implementation of digital health technologies implies new methodological challenges to the standardization of assessment criteria.

If existing Health Technology Assessment methodologies remain unharmonized and interoperable at the EU level, they will be unable to capture the real added value of DHTs. This is what EDiHTA aims to solve to ultimately unlock the potential of DHTs for Europe’s healthcare landscape.

EDiHTA: Innovative Framework for DHT Assessment

EDiHTA aims to be the first digital, flexible, inclusive, validated, and ready-for-use European HTA framework, allowing for the assessment of different DHTs (e.g., telemedicine, mApps, AI) at different TRLs, territorial levels (national, regional, and local), and perspectives (e.g., payer, society, hospital). Implementing a co-creation approach, all relevant stakeholders will contribute to its design, development, and validation. The digital framework will be piloted in real healthcare settings in five major European hospitals and through an open piloting scheme with European DHT developers.

Survey

By participating in this questionnaire, you are helping to identify the most critical factors in the development of new digital health technologies from a technology developer point of view. Your insights are invaluable to advancing the field and ensuring that new assessment methodologies meet the needs of technology developers.

Note: Relevance of each factor should be established on a Likert scale ranging from 1: not relevant/important to 9: very relevant/important.

A. Name of company

B. Country of main market

C. Established (year)

D. Type of technology

1. Mobile App
2. Telemedicine
3. AI
4. Robot
5. Other (please specify?

E. Is there a department of evaluation/assessment (HTA) or Market Access within your organisation?

F. What is the main Therapeutic Area of interest for your company? If more than one please specify the first three.

G. Do you develop all the parts of the technology in house?

1. Clinical need within target population

How important/relevant is the clinical need and target population in your decision to develop the Technology?

2. Size of target population

How important/relevant is the size of the target population in your decision to develop the Technology?

3. Regulatory compliance

How important/relevant are regulatory compliance requirements (besides GDPR) in your development strategy?

4. Time to develop the DHT—Complexity

How important/relevant is the complexity and time required in your decision to develop the digital health technology?

5. Development Costs

How important/relevant are the initial costs to your decision to develop a new digital health technology?

- Device/Hardware maintenance (e.g., shipping costs to and from maintenance location, spare parts)
- Software development/bug maintenance
- Personnel needs (quantity and training)
- Other (please specify)

6. Maintenance costs

How important/relevant is ongoing maintenance costs in your decision to develop a new digital health technology?

- Device/Hardware maintenance (e.g., shipping costs to and from maintenance location, spare parts)
- Software development/bug maintenance
- (quantity and training)
- Other (please specify)

7. **Having a comparator (SoC)**

How important/relevant is having a clear standard of care (SoC) comparator when developing a new digital health technology?

8. **Technical characteristics**

How important/relevant is the adaptability of the technology for future development when initially considering the development strategy of the technology?

9. **Patient safety**

How important/relevant is ensuring patient safety during the use of the technology?

a. In the development phase of the technology
b. In the use of the technology phase

10. **Privacy and security**

How important/relevant is privacy and security of the end user of the technology concerns you before developing the technology**?**

11. **Improvement in clinical outcomes**

How important/relevant are improvements in clinical outcomes for developing a new technology?

- Direct impact on Clinical outcomes
- Integration of care
- Continuity of care (long-term)
- Type of clinical improvement evidence provided using the technology

12. **Readiness of health system (usability and acceptance)**

How important/relevant is the readiness of health systems in the development of your health technology?

13. **Access market timing**

How important/relevant is the market access for your digital health technology?

14. **HTA process (in target market)**

How important/relevant is the Health Technology Assessment (HTA) process in the target market for your development decisions?/

15. **HTA process (in target market)**

Do you investigate the HTA process before making development decisions (Yes/No)?

16. **Pricing and reimbursement process (in target market)**

How important/relevant are reimbursement and market access policies in the target market for your digital health technology development?

17. **Expected Return of Investment (ROI)**

How important/relevant is the expected return on investment (ROI) in your development decisions for digital health technologies?

18. **Readiness of patients (usability and acceptance, adherence)**

How important/relevant is the readiness of patients in the development of your health technology?

- Usability (user experience) of solutions for your target population
- Acceptance and willingness to use the solution for your target population

- Expected adherence for your target population

19. Stakeholder involvement in the development phase

How important/relevant is early involvement of patients/caregivers in the development of the technology?

How important/relevant is early involvement of clinicians/healthcare providers in the development of the technology?

20. Data ownership

How important/relevant is data ownership in your approach to developing new digital health technologies?

21. Data privacy (GDPR)

How important/relevant are data privacy concerns, including GDPR compliance, in your development process?

22. Final comments

Do you have any additional comments or considerations that are important to you when developing a new digital health technology (open question)?

File S3. Focus Group and Interview Protocol

- Introduction of focus group facilitators and participants: Name, Surname, Institution, Role (5 min)
- Introduction of EDiHTA project (5 min)

Challenges and priorities with market access and reimbursement of DHTs

- What are your key challenges when entering multiple European markets at the same time?

  Do you have challenges linked specifically to access to reimbursement?
- What is your approach to market access and reimbursement when entering more European markets at the same time?
- Would you consider assessment of DHTs during different phases of technology development? (e.g., early phase of development, late phase of development, post-market phase)

  How can the assessment framework capture this? How about evidence requirements? Would the developers consider this during development?
- Lack of transparency and predictability on evidence generation requirements for regulatory approval and funding/reimbursement decision-making. How can we take this into account with the EDiHTA framework?

Challenges with specific HTA domains

- What are the key challenges you face with the readiness of health systems and target populations when implementing a technology?
- What are the key challenges you face with privacy, data security, and data ownership?
- Is there a patient-centric approach for developers? Is there a success story or is this not sustainable?
- Is continuous evidence and post-implementation monitoring of DHTs something you could support?
- Any additional comments or considerations that are important to you when developing a new digital health technology?

Vision and Future

- How do you see the future in terms of a common market for DHTs?
- Do you see a common pan-European assessment approach being beneficial to you?

  What would be the best value for you?
- Following the response to the survey and today's discussion:

  What would you expect from this project to provide value to you?

What is the main priority(ies) for you? What kind of information would you like to have through the EDiHTA platform?

- Sustainability of the EDiHTA framework?

  Do you have any further comments?
